# Designing a Logic Model for Mobile Maternal Health e-Voucher Programs in Low- and Middle-Income Countries: An Interpretive Review

**DOI:** 10.3390/ijerph19010295

**Published:** 2021-12-28

**Authors:** Seohyun Lee, Abdul-jabiru Adam

**Affiliations:** 1 Department of Global Public Administration, Mirae Campus, Yonsei University, Wonju 26493, Korea; 2 Department of Public Administration, Mirae Campus, Yonsei University Graduate School, Wonju 26493, Korea; adam2019@yonsei.ac.kr

**Keywords:** maternal health, voucher, mobile e-voucher, mobile money, logic model, LMICs

## Abstract

Despite the increasing transition from paper vouchers to mobile e-vouchers for maternal health in low- and middle-income countries, few studies have reviewed key elements for program planning, implementation, and evaluation. To bridge this gap, this study conducted an interpretive review and developed a logic model for mobile maternal health e-voucher programs. Pubmed, EMBASE, and Cochrane databases were searched to retrieve relevant studies; 27 maternal health voucher programs from 84 studies were identified, and key elements for the logic model were retrieved and organized systematically. Some of the elements identified have the potential to be improved greatly by shifting to mobile e-vouchers, such as payment via mobile money or electronic claims processing and data entry for registration. The advantages of transitioning to mobile e-voucher identified from the logic model can be summarized as scalability, transparency, and flexibility. The present study contributes to the literature by providing insights into program planning, implementation, and evaluation for mobile maternal health e-voucher programs.

## 1. Introduction

Maternal mortality is known as one of the key indicators of health inequity due to its disproportionate occurrence among different socioeconomic groups [1]. According to the World Health Organization (WHO) and the Global Burden of Disease Study 2019, developing countries account for 99% of maternal mortality [1,2]. To minimize probabilities of obstetric complications and maternal deaths that are avoidable, at least four antenatal care (ANC) visits and four postnatal visits are recommended, but the coverage of basic maternal health services varies widely [3,4]. A previous study reported that overall antenatal care coverage in 2013 was 48.1% for developing countries compared to 84.8% for developed countries [5].

These dramatic gaps are expected to persist or may even get worse due to the prolonged COVID-19 pandemic. Specifically, the perceived barriers and the actual limitations in access during the COVID-19 pandemic can result in a low utilization of maternal health services. In Ethiopia, for example, a study involving 389 pregnant women reported 55.5% late or missed ANC visits during the COVID-19 pandemic period and, of those, 56% were due to fear of COVID-19 infection and 33% were due to interrupted maternal services [6]. According to COVID-19 surveys from India and DR Congo, about 50% of women had difficulties in accessing health facility during COVID-19 restrictions [7].

More importantly, the unmet maternal health needs in low-resource settings have been largely attributed to lack of financial resources. For example, the Performance Monitoring for Action survey from Kenya found that 91% of the female respondents lost household income partially or completely since COVID-19 restrictions, which could lead to the worst case scenario in terms of maternal health care service utilization [7]. In fact, financial constraints have been considered as one of the main causes for insufficient supply and demand for maternal health services in the past years [8]. A systematic review on access barriers to obstetric care in sub-Saharan Africa identified that the principal barrier was limited household resources or income [9]. Recent reports from the Demographic and Health Survey conducted during 2019 and 2020 in Liberia, Gambia, or Sierra Leone found that the most frequently mentioned barrier for women to get access to health services was getting money for treatment [10,11,12]. Another empirical study on access to healthcare among 307,611 sub-Saharan African women also pinpointed that the predominant barrier was money [13].

To tackle these maternal health disparities, huge financial contributions have been made over the past decades in the form of foreign aid or development assistance. Particularly, the maternal health issue has been given much attention as one of the health focus areas for development assistance since the Millennium Development Goals (MDGs) era. Furthermore, a previous study provided empirical evidence for the growing development assistance for maternal and child health since 2010, compared to other health focus areas such as HIV/AIDS, which showed a flattened or downward curve [14]. Given the fact that cost-effective maternal health services have been unequally available in many parts of the world, donors have used a number of innovative approaches to deliver these services for the underserved populations. One of the interventions to overcome financial barriers is results-based financing. Results-based financing is an umbrella term for a mechanism that provides financial incentives to the provider or service users only if pre-defined results are achieved [15].

There are two different approaches to results-based financing—supply-side and demand-side—depending on the focus of the scheme [16]. Supply-side financing offers incentives to providers, whereas demand-side financing provides subsidies for services or merit goods directly to the beneficiaries [17]. A voucher program is one of the methods for demand-side financing, which has been employed to remove barriers to accessing various health services, especially maternal and reproductive health. The rationale behind the maternal health voucher programs is that it can encourage care-seeking behavior by alleviating the burden of costs associated with maternity care services. Maternal health voucher programs have been implemented in low- and middle-income countries (LMICs) such as Kenya, Uganda, Pakistan, and Bangladesh. Evidence on the effectiveness of these programs has also been reported in previous studies, which mostly demonstrated the increase in maternal health service utilization after the implementation of voucher programs [18,19,20,21].

Recently, an electronic voucher or e-voucher involving mobile phone systems for maternal health has been experimented as an alternative to physical tokens or paper coupons. Mobile e-vouchers have the potential for improving the current operational process in various ways, such as real time tracking and monitoring or emergency communication. Transition from paper vouchers to mobile e-vouchers can allow transparency for reducing fraud and flexibility by adding or adjusting included services. A plastic card that contains bar codes is one option to deliver e-vouchers, but mobile app or SMS-based e-vouchers can be more feasible in LMICs where mobile subscription rates are getting higher. In addition, mobile money has been widely used in the daily lives for many sub-Saharan African countries such as Ghana, Kenya, or Tanzania. In Kenya, for example, a mobile maternal health e-voucher program integrated mobile money for providing maternal health services along with transportation cost subsidies [22].

As the mobile e-voucher system is a new approach with short history, little is known about the best practices or implementation strategies in the context of LMICs. For instance, a recent report on e-vouchers for family planning services addressed that no data were found in terms of comparative effectiveness between e-voucher and paper vouchers, or the impact of e-vouchers on health outcomes [23]. In this context, this study aimed to design a logic model for mobile maternal health e-voucher programs using an interpretive review approach. In the first step, evidence was synthesized from both the traditional and mobile e-voucher programs for maternal health in LMICs through an interpretive review approach. Based on this evidence, key components required to design a mobile e-voucher program for maternal health were synthesized and suggested within a logic model framework. For decades, logic models have been used for communicating a program cycle ranging from planning, implementation to monitoring, and evaluation [24]. In doing so, this study will provide recommendations for a successful transition from paper to mobile e-voucherd for maternal health services in LMICs.

## 2. Methods

### 2.1. Search Strategy and Review Process

To identify the studies on maternal health voucher programs in the context of LMICs, electronic databases (MEDLINE through Pubmed, EMBASE, and Cochrane Database of Systematic Reviews) were searched. The review question for this interpretive evidence synthesis is as follows: “What are the programmatic elements that should be considered to develop and evaluate a mobile maternal health e-voucher programs in LMICs?”. After several trials for finalizing the optimal search terms, “maternal” and “voucher” were chosen to achieve the balance between the sensitivity and specificity of the search results [25]. In other words, only two representative terms for the population and intervention parts of the conventional PICO format for a systematic review were built into the search strategy. No restrictions were applied to the country or publication type, although studies on high-income countries were to be excluded during the screening process. Additional search was performed from the grey literature and references of the previous review studies. The search included articles and reports published up until May 2021.

The authors (S.L. and A.j.A.) independently reviewed the retrieved articles and reports based on the titles and abstracts. After removing duplicates and irrelevant studies from this first stage, a full-text review was conducted. The disagreement during the selection process was resolved by a discussion between the two authors.

### 2.2. Eligibility Criteria for Review

The following inclusion criteria were used for this interpretive review. First, the study population should be defined as women of all ages who were either pregnant or within 42 days of the conclusion of pregnancy for the postnatal period, as defined by the WHO [26]. Second, the study should deal with a voucher program for maternal health services in LMICs, as defined by the World Bank’s classification by income level [27]. Third, the study should provide implications regarding inputs, activities, outputs, and outcomes of the voucher program. Fourth, the study should be published in English.

Studies were excluded for the following conditions. First, if the full-text was not available or there was only an abstract for conference presentation, the study was excluded. Second, studies that discussed voucher programs for goods or services that were not directly related to maternal health were excluded. Third, review articles were excluded, but the individual studies identified from the relevant reviews that met the inclusion criteria were included for the evidence synthesis.

### 2.3. Data Extraction and Synthesis

After reviewing the retrieved studies based on the inclusion and exclusion criteria, the information about the setting, target population, and included services under the voucher scheme were extracted. Specifically, key programmatic elements for the inputs, activities, outputs, and short-term and long-term outcomes were analyzed and transcribed into the logic model framework. As discussed in a previous study that used the systematic review approach for building a logic model, each element for the program planning, implementation, monitoring, and evaluation was categorized into the framework [28]. The logic model was developed under the assumption that the key elements identified from the traditional maternal health voucher programs, as well as mobile e-voucher programs, should be considered for a mobile maternal health e-voucher programs, because the fundamental structure or cycle of the program would not be drastically changed by the transition from paper-based voucher to mobile e-vouchers [23]. The developed logic model was cross-validated with another logic model that was recently published as part of a protocol for a mobile maternal health e-voucher program in Cameroon [29]. This study is the only published protocol with a logic model so far.

### 2.4. Quality Appraisal

Conventional systematic review studies that aim for finding evidence of effectiveness or testing a theory usually assess the quality of the included studies either by (1) including only a certain study designs (e.g., randomized controlled trial) or (2) using structured quality assessment tools for a specific study design. However, these approaches are not feasible for the scope and purpose of our study, because the goal is to give insights into program design and evaluation by including various types of relevant publications with sufficient evidence, regardless of the study design. For example, the studies identified in this review include reports or qualitative studies that are generally excluded in conventional systematic reviews. Therefore, we employed suggestions by Dixon-Woods et al., which discussed a quality assessment method for maximizing the inclusion and contribution of diverse empirical studies [30]. This approach was originally recommended by the National Health Service of the United Kingdom and was adapted for an interpretive review. The five criteria for informing judgements about quality of studies are as follows [30].

- Are the aims and objectives of the research clearly stated?
- Is the research design clearly specified and appropriate for the aims and objectives of the research?
- Do the researchers provide a clear account of the process by which their findings we reproduced?
- Do the researchers display data to support their interpretations and conclusions?
- Is the method of analysis appropriate and adequately explicated?

Each of the above five appraisal questions were scored 1 if yes, and 0 if no. The maximum total score was 5 and the minimum was 0. However, no studies were excluded on the basis of the quality appraisal score.

## 3. Results

**Figure 1:**
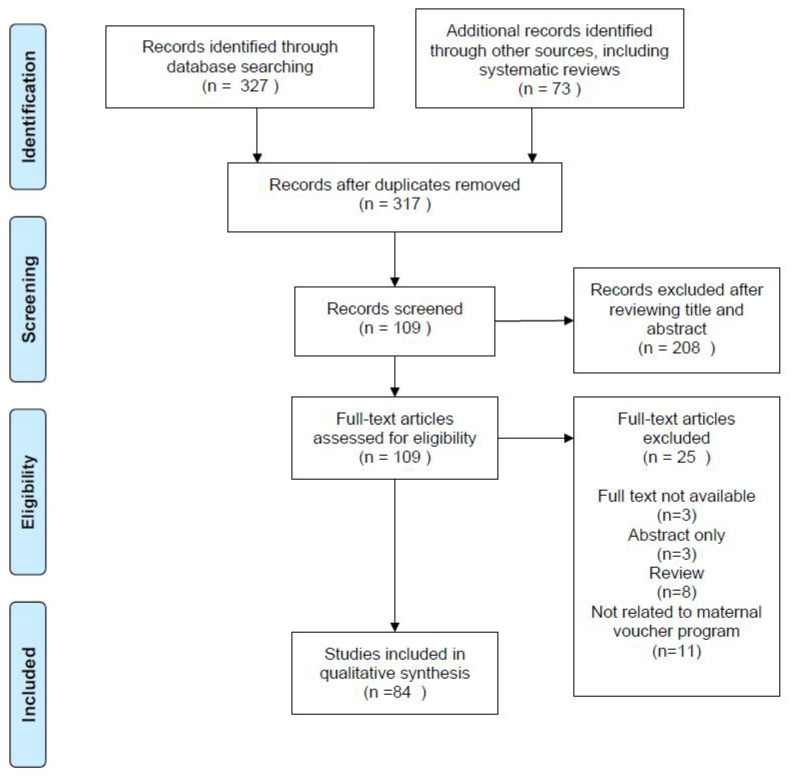
Flow diagram for selection process following the Preferred Reporting Items for Systematic Reviews and Meta-Analyses (PRISMA) guideline.

A total of 317 studies were identified and screened for the title and abstract. Of those, 208 were excluded and 109 articles were reviewed. Among 109 studies, 25 were excluded for the following reasons. First, three conference abstracts and three studies without full-text were excluded. Second, eight review articles were not included, but their references were screened thoroughly to identify any eligible studies to be included in the analysis. Third, 11 studies not related to the maternal voucher program for pregnant women were excluded, such as family planning vouchers for women with HIV or urban youth [31,32]. Therefore, 84 studies were included for interpretive synthesis of the evidence. A flow diagram for the selection process, based on the Preferred Reporting Items for Systematic Reviews and Meta-Analyses guidelines, is illustrated in Figure 1.

### 3.1. Overview of Included Studies

Among the 84 studies, 49 discussed maternal health voucher programs in sub-Saharan Africa and 35 were about the programs in Asia or the Middle East. Specifically, studies from Uganda, Cameroon, Kenya, Tanzania, Laos, Myanmar, Indonesia, Cambodia, Yemen, Bangladesh, India, and Pakistan were included for the analysis. A total of 27 maternal health voucher programs (14 for sub-Saharan Africa and 13 for Asia or Middle East) were identified—most of which were discussed in multiple studies, while Jordanwood et al., 2021, Bellows et al., 2012, and Arur et al., 2009 introduced two separate programs in their studies [33,34,35]. Summaries of the included studies are presented in Table 1 and Table 2.

The benefits package for the maternal health voucher programs can be divided into three components: maternal health services or products, transportation, and communication. First, maternal health services that were covered under the voucher scheme generally included four ANC visits, institutional delivery, and one PNC visit. For some of the programs in Pakistan, India, and Cambodia, three ANC visits instead of four were specified under the voucher scheme. In addition, maternal health-related products such as sanitary products for clean and safe delivery were provided under some voucher programs. Second, the transportation costs were covered either for emergency referral only or travel for any maternal health-related visits. The transportation support was provided as a stand-alone voucher program or in combination with maternal health services or products. Third, communication was one of the important components for maternal health voucher programs, in that it allowed for behavior change communication and emergency contacts. The communication component was enabled by the mobile phone system that was introduced as part of the mobile phone credit voucher program or mobile maternal health e-voucher programs.

Fourteen programs (51.9%) involved both maternal services and transportation. Five programs (18.5%) offered transportation alone, while four programs (14.8%) covered only maternal services or products. One program (3.7%) provided mobile phone credit vouchers for communication. Three programs (11.1%) that employed a mobile money platform or e-vouchers had benefits package with all three components—maternal services or products, transportation and communication.

As for the quality appraisal of the included studies, each study was scored based on the five appraisal questions suggested by Dixon-Woods et al. The quality appraisal scores ranged from 1 to 5, and the average score was 4.34 (SD 0.97) and 4.68 (SD 0.55) for Asia or the Middle East and sub-Saharan Africa, respectively.

#### Studies Involving Mobile Phone System

Out of 14 maternal health voucher programs in sub-Saharan Africa, seven (50%) programs involved a mobile phone system. Two programs in Uganda adopted the mobile phone system midway through the implementation [35,116], whereas three programs in Kenya were initially designed for using mobile money or a mobile e-voucher platform [75,76,78]. One study from Cameroon was discussed as a pilot protocol for the mobile e-voucher [29] and another program in Tanzania provided mobile phone credit vouchers for direct two-way communication between pregnant women and healthcare providers [79,80].

These programs can be categorized into four different types. First, programs in Uganda used the mobile phone system for operational process, such as reimbursement via mobile money for the drivers or electronic claims submission during the COVID19 pandemic. Second, programs in Kenya provided e-vouchers to the pregnant women via already existing mobile money system and sent reminder text messages. Third, a mobile application was developed for the program in Cameroon to offer e-vouchers and two-way communication. Fourth, a program in Tanzania empowered pregnant women with a mobile phone credit voucher so that they can communicate with healthcare providers in a timely manner.

### 3.2. Logic Model for Mobile Maternal Health e-Voucher Program

By synthesizing the evidence from the literature on the traditional maternal health voucher programs, as well as those involving the mobile phone system, the key elements for inputs, activities, outputs, and short-term and long-term outcomes were identified. The overview of the logic model for a mobile maternal health e-voucher program is presented in Table 3, Table 4 and Table 5. The elements that are directly related to mobile phone system appear in bold text. As the included studies suggest, the benefits package of a maternal health voucher program consists of three components: (1) maternal health services or products, (2) transportation, and (3) communication support. However, it should be noted that the logic model largely focused on maternal health services or products, given that a majority of the programs (77.8%; 21 out of 27) mainly covered maternal health services or products under the scheme. For this reason, the logic model for each of the three components was developed and presented in Table 3, Table 4 and Table 5, but the specific descriptions below focus only on the maternal health services and products in Table 3. Various elements were identified for multiple times in different studies, but presented without duplication in the logic model framework.

#### 3.2.1. Inputs

The elements for inputs were grouped and organized into the following five categories: (1) infrastructure and system, (2) organization, (3) staffing, (4) funding and resources, and (5) tools.

First, both traditional and mobile e-voucher programs require basic infrastructure and system such as health information management system or electricity. For mobile e-voucher programs, mobile money platforms or mobile applications were needed as additional inputs.

Second, examples of organization elements included the voucher management agency (VMA), contracted facilities, and independent audit or evaluation agencies.

Third, key elements for the staffing were voucher distributors, community health workers, volunteers, study coordinators, and project managers. In the case of mobile e-voucher programs, community health workers or voucher distributors should be provided with support for mobile communication.

Fourth, in terms of funding and resources, overall funding for the program and the financial resources for various incentive mechanisms were identified. The examples of such mechanisms included conditional cash transfer for mothers with four ANC visits, financial support for volunteers’ travel, a small premium to compensate facilities for administrative burden, and incentives for community health workers for identifying beneficiaries.

Fifth, as for the tools, the voucher, basic supplies and equipment, poverty grading tool for means testing, treatment guidelines, and facility assessment tool for contracting and accreditation were identified. Additionally, a theoretical framework for program design was suggested as a key tool.

#### 3.2.2. Activities

Activities required for a mobile maternal health e-voucher program can be organized into six categories: (1) program design; (2) sensitization; (3) training, workshop, and mentorship; (4) payment; (5) implementation; and (6) monitoring and evaluation.

First, preparatory activities were required for the program design. The examples included formative research, birth rate estimation, and revenue planning. In addition, benefits package design, selection of the target and eligibility criteria, review on current standard MCH services, field visits, selection of providers, development, and needs assessment of the communication strategy were performed in the previous voucher programs. Another important element was the design of a health information management system, which can involve electronic systems.

Second, activities related to sensitization were identified as follows. The methods for sensitization were diverse, such as radio, drama, posters, events, or multimedia campaigns. The targets for the sensitization were community leaders, school, church, traditional healers, or political leaders. Additionally, the mobilization of local NGOs and community health workers, and home visits for voucher promotion, could be considered.

Third, training, workshop, and mentorship were key elements for the most voucher programs. The targets for the training included health workers, midwives, community health workers, and so on. Monthly group meetings, mentorship, or training via mobile applications were also suggested in the published literature.

Fourth, payment was one important process for a voucher program. The activities for the payment involved reimbursement, verification of vouchers, claims processing, fraud control, adjustment of salaries or reimbursement rates, and cost minimization evaluations. Optional elements included transportation subsidy payments made by health centers and accommodation of installment payment.

Fifth, activities for implementation included those required in the early stage and those that happen at a later stage. The activities for the early-stage implementation were preparation of facilities, dissemination of project information, engagement of traditional birth attendants, pilot test, accreditation, and contracting. Once these activities were completed, the identification, screening and enrollment of the participants, distribution or sale of vouchers, quality assurance, communication with providers, comorbidity assessment, stratification and/or randomization, and project management should be carried out. In addition, health education and promotion activities, operation of one stop shops, scale up planning and implementation, and cluster randomized controlled trials can be considered.

Sixth, activities related to monitoring and evaluation involved supervision, audits, surveillance, and registry reviews. For a follow-up with clients, various activities were performed such as pre- and post-surveys, client satisfaction surveys, field testing of data collection forms, data collection and geo-mapping via mobile phone, validation of claims, verbal autopsy on maternal deaths, and survey by an independent market research agency. In addition, health technology assessment, cost-effectiveness analysis, evaluation of midwives, and delisting of facilities were conducted in some voucher programs.

#### 3.2.3. Outputs

Key elements for the outputs were categorized as follows: (1) tools, (2) staffing, (3) payment, (4) implementation, (5) facilities, and (6) voucher use.

First, outputs related to tools included manuals, reports, guidelines, patient-held record, reporting templates, and anti-fraud policies. Additionally, the number of supplies or equipment provided or the average number of equipment were considered as outputs for the tools.

Second, outputs for the staffing were the number of staffs, newly hired providers, and staffs trained, or the percentage of facilities with at least one doctor. In addition, the average number of human resources for the participating facilities were suggested.

Third, outputs related to payment were provider payment rates, time from invoice to payment, midwives’ monthly income, and unofficial payments regardless of voucher use.

Fourth, the number of meetings, marketing activities, or trainings and timeliness of planned activities were considered as outputs related to implementation. Additionally, the number of pregnant women who received sensitization campaigns or who were able to activate GIS feature and feedback into the mobile platform were considered in a mobile maternal health program.

Fifth, outputs for the facilities were identified in the context of accreditation and quality. Examples included the number of accredited facilities, facilities that were improved, or districts covered under the voucher program. Those related to quality improvement were the percentage of facilities with an adequate infrastructure, facilities offering 24/7 services, or facilities with quality improvement activities.

Sixth, outputs related to voucher use included the number of vouchers distributed, sold, used, refunded, fraud cases, or voucher claims submitted. In addition, the percentage of participants who completed follow-up or who do not follow procedures as allocated were considered for the experimental studies. Other examples included the percentage of contamination; percentage of eligible women who received voucher; average length of follow-up; the number of women identified, contacted, and registered; the number of visits to the villages; and increased demand or coverage.

#### 3.2.4. Short-Term Outcomes

Short-term outcomes were (1) service utilization—delivery; (2) service utilization—ANC, PNC, and others; (3) staff and resources; (4) women’s experience; (5) quality; (6) costs; and (7) competition and governance.

First, the service utilization outcomes related to delivery were frequently mentioned in the included studies. Examples of such outcomes were the proportion of estimated deliveries by the voucher, percentage or number of institutional deliveries, number of deliveries by skilled birth attendants, and percentage of home delivery.

Second, the service utilization outcome for the ANC, PNC, and other services involved the proportion of women with at least four, three, or two ANC visits; PNC visits; the number of each service delivered by the voucher; or increased equity in service utilization. In addition, gestational age at first ANC visit, the number of women who completed the sequence of services, and the percentage of women who sought care within 1 h from the onset of symptom were considered.

Third, the outcomes related to staff and resources investigated the job satisfaction in terms of workload, salary and staffing, challenges in identifying beneficiaries or distributing vouchers, effects on workload, and providers’ attitudes toward the program. In addition, motivation of providers for higher quality service and changes in the resource use were included.

Fourth, women’s experiences were considered as one of the important short-term outcomes. These outcomes were aimed to recommend the voucher to a friend, awareness of the program, the facilitator, barriers or reasons for voucher use, and challenges faced by the women. Additionally, women’s decisions on having more children, proportion of births prepared, perceived barriers, knowledge, and attitude and practice towards the MCH services were discussed.

Fifth, the quality outcomes addressed facility level issues such as percentage of facilities that adequately considered medical history, created rapport, and prepared for equipment and supplies. In addition, service waiting hours, round-the-clock service availability, increased efficiency in service delivery, and knowledge and skills of providers were suggested. The quality outcomes were also explored from the clients’ perspectives, such as satisfaction with overall experience, respect shown by the staff, perceived quality, perception on contracted facilities and quality of care, or privacy issues.

Sixth, the outcomes related to costs included out-of-pocket expenditure, average total cost, reimbursement costs or weighted average costs for each service, and incremental cost per institutional delivery. Other options were explored in the included studies, such as percentage with additional emergency cost, costs of setup and intervention, protection from financial catastrophe, or net cost per family. Key elements for cost-effectiveness studies were also examined, such as direct medical costs, direct non-medical costs, indirect costs, total cost of base wages, or total direct financial assistance to beneficiaries.

Lastly, competition and governance outcomes were explored in the included studies, given that the voucher programs in principle are intended to encourage competition among providers and better governance by empowering the clients [116]. The examples included competition among providers, increased client choices, bypassing of low-quality service, checks and balance mechanisms, accountability, and opportunities for learning and adapting to local settings.

#### 3.2.5. Long-Term Outcomes

Long-term outcomes identified from the studies included (1) maternal and neonatal health outcomes, (2) cost-effectiveness, (3) sustainability of the program, and (4) integration into the health system. Among these, maternal and neonatal health outcomes were most extensively discussed in the included studies. For example, neonatal mortality, infant mortality, maternal mortality, institutional perinatal mortality, and still birth rates were key elements for long-term outcomes. In addition, outcomes related to complications such as post-partum hemorrhage, obstructed labor, or birth asphyxia were examined. Furthermore, maternal and neonatal health outcomes for cost-effectiveness evaluations were explored, such as deaths averted, DALYs averted, or life years saved per 1000 vouchers distributed. In addition to these maternal health outcomes, the cost-effectiveness outcomes were investigated, such as cost per death averted or life year saved, and cost per DALY averted. Other long-term outcomes included sustainability of the program and integration into the existing health system.

### 3.3. Cross-Validation of the Logic Model

**Figure 2:**
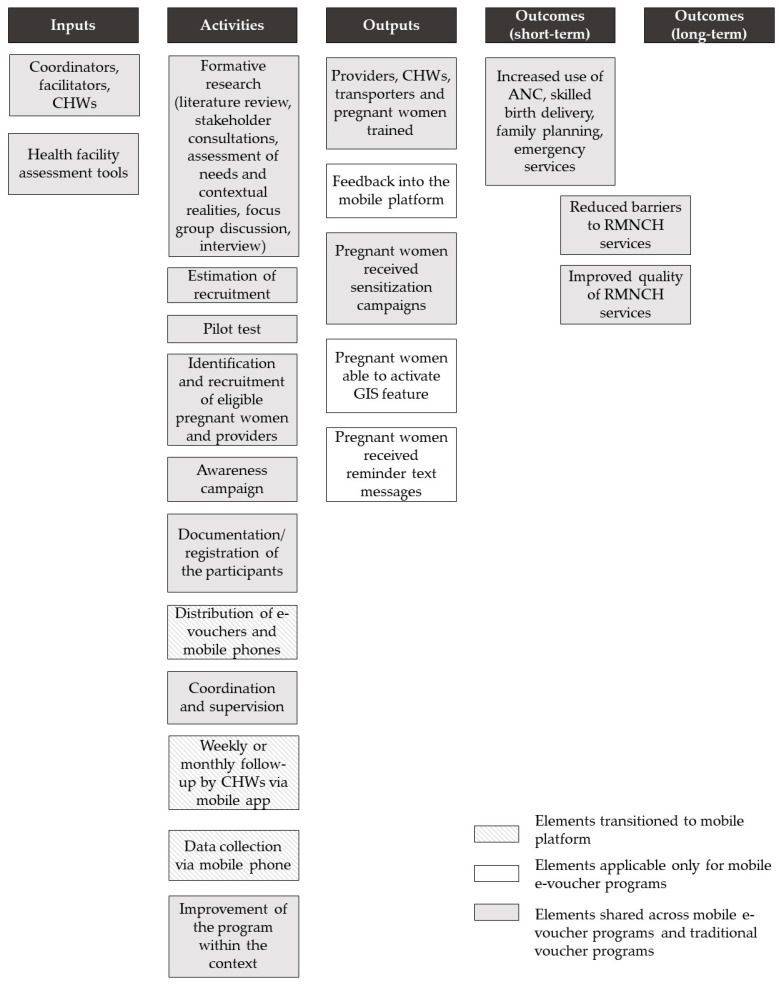
Overview of the published logic model from the mobile maternal health e-voucher program in Cameroon for cross-validation.

For cross-validation purposes, the logic model developed from this interpretive synthesis of evidence was compared with a logic model published in a previous study on the mobile maternal health e-voucher program in Cameroon [29]. All the elements identified from their study were already included in the logic model developed from this systematic review. Figure 2 shows the overview of the logic model from the Cameroon study and the results of cross-validation. Their study did not include long-term outcomes due to the relatively short (18-month) study period. Most of the elements were shared across the traditional paper-based voucher programs and mobile e-voucher programs. Other elements such as feedback into the mobile platform, activation of the GIS feature, and reminder text messages were applicable only for mobile e-voucher programs. In addition, elements that were transitioned to mobile platforms were identified. Examples of such elements included the distribution of e-vouchers and mobile phones, instead of paper-based vouchers, follow-up contact, and data collection via mobile applications.

## 4. Discussion

By conducting an interpretive review of maternal health voucher programs in LMICs, this study aimed to develop a comprehensive logic model for mobile maternal health e-voucher programs. Building upon the previous literature on both traditional voucher programs and mobile e-voucher programs, this interpretive evidence synthesis suggests a logic model that can be utilized in a real-world setting. A total of 27 maternal health voucher programs from 84 studies were thoroughly reviewed to identify key elements for inputs, activities, outputs, and short-term and long-term outcomes. Among these 27 programs, 7 programs involved mobile phone system for various purposes, including claims processing, payment for the service providers, mobile phone credit for communication between pregnant women and the providers, mobile e-vouchers, or mobile money. The elements within a logic model were categorized and organized into themes so that the final logic model is presented in a systematic way.

Although several review studies on maternal health voucher programs have been published in the last decade, none of them deal with mobile maternal health e-voucher programs. In this context, the present study contributes to the literature by discussing mobile e-voucher programs for LMICs where the average mobile subscription rates per 100 population was 104.7 in the year 2020 [117]. Additionally, mobile money has already proliferated many LMICs and changed the mechanism for financial transactions in everyday life [118]. Given this situation, the mobile e-voucher programs have already been started in a few LMICs such as Cameroon or Kenya [29,78]. By taking advantage of the most popular mode of telecommunication in LMICs, the mobile maternal health e-voucher programs can still demonstrate the proven effectiveness of the traditional voucher programs while improving their efficiency.

Despite its potential, mobile e-voucher programs may face high startup cost, such as investment in hardware, development of software systems, or additional training for the staff [23]. However, the long-term return on investment can be higher than the traditional paper voucher programs, as the costs per client will decline by saving on the administrative costs of printing and distribution, fraud control, monitoring, and claims processing and payment. For example, a previous study on a traditional maternal voucher program in Uganda suggested the use of mobile phones for making payments to reduce transaction costs [41]. As suggested in the logic model, payment is one of the important activities that can greatly improve efficiency by shifting to mobile e-vouchers.

The benefits of transitioning to mobile e-vouchers identified from the logic model can be summarized as scalability, transparency, and flexibility. First, a mobile e-voucher program has a comparative advantage in scaling up. As in the case of the Bangladesh maternal voucher program, most maternal health voucher programs indeed started with only a few districts and then scaled up to cover larger geographic areas [94]. To reach scale, keeping management costs low is essential, which can be achieved by alleviating the administrative burden [16]. In this sense, a mobile maternal e-voucher program can be a viable option because it eliminates the administrative processes of printing, stocking, and physically distributing the vouchers, which were identified as key elements in the logic model. Additionally, mobile e-voucher systems can simplify the data entry for registration and client management.

Second, transparency in voucher management can be improved in mobile e-voucher programs by electronically tracking and following up with voucher utilization and redemption. As presented in the logic model developed from this study, the follow up activities and fraud control policy were emphasized. In fact, studies argued that the fraudulent activities or fabricated voucher claim forms are the most frequently cited concerns in traditional voucher programs, so additional investment had to be made for security features on printed vouchers, such as watermarking [17,23,119]. With a mobile e-voucher, however, general program cycle from enrollment to claims processing and payment can become more transparent by allowing for real time cross-checking and tracking.

Third, mobile e-vouchers are more flexible in terms of adjusting reimbursement rates or included services. After the rollout, maternal health voucher programs may undergo adjustments in reimbursement rates to attract more providers, as discussed in a previous study in Uganda [34]. In addition, some of the services were newly added or excluded midway through the implementation, as evidenced by the voucher program in Uganda [45]. Unlike traditional paper voucher programs, the changes resulted from these adaptations can take effect immediately, with little confusion for mobile e-voucher programs. Furthermore, mobile e-voucher programs can deliver several combinations of services efficiently. As presented in the logic model developed by the present study, a maternal voucher program generally covers three key components as the benefits package—namely, maternal health services or products, transportation, and communication. The transportation component is an integral part of maternal health voucher programs in LMICs, as suggested by previous studies that reported the women’s financial burden for travel costs [120]. In addition, the communication enabled by a mobile phone system can be integrated into the voucher scheme to promote behavior change communication, emergency calls, or GIS feature. All these components can be effectively managed electronically if a mobile e-voucher program is implemented.

In fact, electronic voucher schemes have been implemented in several high-income countries as well. For example, an electronic voucher program for maternal health services was launched in South Korea in 2008 [121]. The maternal e-voucher program offers approximately USD 500 worth of maternal health services for all pregnant women under the universal health insurance scheme in South Korea. A previous study reported the reduced risk of preeclampsia after the introduction of this universal voucher scheme for maternal health [122]. Another example of e-vouchers for maternal and child health in a high-income country is the Special Supplemental Nutrition Program for Women, Infants, and Children, also known as the WIC program, in the United States. A recent study showed that beneficiaries had a positive attitude towards the transition to the electronic benefits transfer (eWIC) system, which involves electronic redemption of the food items [123].

This interpretive review identified the studies on maternal health voucher programs in LMICs and synthesized evidence to develop a logic model for a mobile e-voucher program for maternal health. Although key elements for the logic model were thoroughly reviewed and presented in this study, there are still a few limitations. First, only studies published in English were included for analysis. As a result, maternal health voucher programs that did not have any publications in English could not be accessed. To minimize the risk of missing any valuable information about the programs, this study included all types of publications such as reports or issue briefs, unlike the traditional systematic review approach that generally considers only the specific study design. Second, this study could have provided more practical information if it involved voices from the stakeholders. As part of vetting process for the logic model, future research can be conducted along with stakeholder consultations. Third, a small number of studies on mobile e-voucher programs were identified and included for analysis, even though the purpose was to develop a logic model that suggests a transition from paper to mobile e-vouchers. As the uptake of the e-voucher approach is growing in other areas such as agriculture, family planning, and insecticide-treated bed nets, the application in maternal health will also likely to increase in the near future [23]. In this sense, opportunities will be available for a future systematic reviews that can rigorously assess the risk of bias of studies on mobile maternal health e-voucher programs.

## 5. Conclusions

Maternal health vouchers have been introduced in many LMICs over the past decade, and previous literature confirmed the effectiveness of such programs for increasing the utilization of maternal services for the underserved women. This interpretive review attempted to take it one step further so as to provide evidence for the recently growing mobile e-voucher programs. In doing so, key elements for the inputs, activities, outputs, and short-term and long-term outcomes were identified and systematically organized to develop a logic model. The findings from this study suggest that the majority of elements overlap between traditional paper vouchers and mobile e-vouchers. For example, long-term outcomes that should be achieved are reductions in maternal and neonatal mortality, regardless of the voucher type. However, some of the elements have the potential to be greatly improved by transitioning from paper to mobile e-vouchers. By presenting the overview of an evidence-based logic model for mobile e-voucher programs for maternal health, this study provides insight into the planning, implementation, and evaluation of the program.

## Figures and Tables

**Table 1:** Summary of included studies for sub-Saharan Africa.

Country	Program	Target Population	Included Services under Voucher Scheme	Author, Year	Quality ^1^
Uganda	“Healthy Baby” voucher funded by German Development Bank (KfW) and the Global Partnership on Output-Based Aid (GPOBA-World Bank)	(Southwestern Uganda) Women who are from voucher program districts and meet the poverty criteria assessed by a poverty assessment tool	Maternal services and transportation: A voucher can be purchased for USD 1.50, which covers (1) 4 ANC visits, (2) transport in case of emergency, (3) treatment for complications and referral, and (4) PNC for up to 6 weeks	Arur et. al., 2009 [34]	4
				Bellows et al., 2012 [33]	4
				Reproductive Health Vouchers Evaluation Team, 2012 [36]	4
				Kanya et al., 2014 [37]	5
				Okal et al., 2013 [38]	5
				Brody et al., 2015 [39]	5
				Obare et al., 2016 [40]	5
Uganda	Demand- and supply-side incentive program by Makerere University College of Health Sciences (MakCHS)	(Eastern Uganda) Pregnant women who are residents of 2 districts of Kamuli and Pallisa	Maternal services and transportation: Pilot phase: A voucher booklet is distributed, which contains 12 transport vouchers (flat rate of USD 2.5 per trip) and 7 service vouchers for ANC, delivery, and PNC Implementation phase: A voucher booklet is distributed during ANC visits and included (1) delivery care, (2) PNC for high-risk mothers or babies, and (3) transportation to health facilities (ranging from USD 1.5 to 2.5 per trip)	Pariyo et al., 2011 [41]	4
				Ekirapa-Kiracho et al., 2011 [42]	5
				Mayora et al., 2014 [43]	5
				Alfonso et al., 2015 [44]	5
				Bua et al., 2015 [45]	5
				Timsa et al., 2015 [46]	5
Uganda	Transport voucher within MANEST ^2^	(Eastern Uganda) Pregnant women in 1 district in Eastern Uganda	Transportation: Transport vouchers for institutional deliveries either (1) fully paid for the poor or (2) partially paid for all pregnant women (reimbursement for the drivers was initially made in cash but evolved to mobile money system)	Namazzi et al., 2013 [47]	5
				Paina et al., 2019 * [48]	4
Uganda	“Boda boda” (motorcycle) vouchers and private service vouchers under SMGL ^3^	Pregnant women in selected rural districts of Uganda	Maternal services and transportation: Free transport vouchers and vouchers for private care	Kruk et al., 2013 [49]	5
				Healey et al., 2019 [50]	4
				Serbanescu et al., 2019 [51]	5
				Conlon et al., 2019 [52]	5
				Ngoma et al., 2019 [53]	5
Uganda	Transport vouchers and baby kit by Doctors with Africa CUAMM (NGO)	Pregnant women in the Oyam District	Transportation: A transport voucher (flat rate of USD 4) was given during ANC visits to cover travel costs for any pregnancy related conditions, emergency, or delivery	Massavon et al., 2017 [54]	5
				Massavon et al., 2019 [55]	5
Uganda	Second Uganda reproductive health voucher project (URHVP) funded by the World Bank	Poor pregnant women in 28 districts	Maternal services: A voucher can be purchased for USD 1.10, which covers (1) 4 ANC visits, (2) a normal delivery, (3) 2 PNC visits, and (4) a choice of an implant for postpartum family planning	Jordanwood et al., 2021 * [35]	5
Uganda	Uganda voucher plus activity (UVPA) funded by the USAID	Poor pregnant women in 35 districts	Maternal services: A voucher can be purchased for USD 1.10, which covers (1) 4 ANC visits, (2) a normal delivery, (3) 2 PNC visits, and (4) a choice of an implant for postpartum family planning (claims submitted electronically or via SMS after the COVID19 pandemic)		
Kenya	Reproductive health Output-Based Aid Voucher program (Voucher for Health) by the Government of Kenya, the Federal Ministry of Economic Cooperation and Development (BMZ), and the KfW	Poor women in 5 Kenyan districts, namely, Kiambu, Kisumu, Kitui, Korochogo, and Viwandani; 2 districts (Kiliff and Kaloleni) were added in the second phase, while in phase 3, Kaloleni were excluded from the target area	Maternal services and transportation: Three vouches can be purchased at USD 1.82, 0.91, and 0 (free), which cover (1) safe motherhood (SMH), namely, 4 ANC visits, institutional delivery care, including caesarean section and medical complications, ambulance transfer to referral facility, 1 PNC visit up to 6 weeks post-delivery; (2) family planning (FP), namely, IUCD, hormonal implants, and sterilization/vasectomy; and (3) gender violence recovery service (GVRS), namely, molestation, rape, and special rape cases	Arur et. al., 2009 [34]	4
				Janisch et al., 2010 [56]	4
				Abuya et al., 2012 [57]	5
				Bellows et al., 2012 [33]	4
				Warren et al., 2011 [58]	5
				Population council 2011 [59]	5
				Armstrong 2012 [60]	3
				Bellows et al., 2013 [61]	5
				Amendah et al., 2013 [62]	5
				Obare et al., 2013 [63]	5
				Obare et al., 2015 [64]	5
				Kumar et al., 2013 [65]	4
				Njuki et al., 2013 [66]	5
				Obare et al., 2014 [67]	5
				Kihara et al., 2015 [68]	5
				Warren et al., 2015 [69]	5
				Watt et al., 2015 [70]	5
				Njuki et al., 2015 [71]	5
				Oyugi et al., 2018 [72]	5
				Dennis et al., 2018 [73]	5
				Dennis et al., 2019 [74]	5
Kenya	Samburu Maternal Neonatal Health Project Phase II (Mobile money-based transport vouchers) by M-PESA Foundation	Women in their third trimester of pregnancy in Samburu Kenya	Transportation: Mobile money-based transport voucher for facility delivery for mothers who have access to mobile phones with a Safaricom SIM card	Ommeh et al., 2019 * [75]	5
Kenya	Maternal Voucher (“m-Kadi” program)	Pregnant women in western provinces (Vihiga County)	Maternal services, transportation and communication: Three intervention types with two treatments and a control, yielding 3 × 3 × 3 design: (1) Full voucher or co-pay voucher (USD 1.2 via mobile money), which covers ANC, delivery, PNC and transportation for referral cases (2) Unconditional cash transfer or conditional cash transfer via a mobile money platform for ANC, PNC, and delivery (3) Weekly reminder text messages (plain or contextualized version)	Grepin et al., 2019 * [76]	4
Kenya	Integrated ANC program by the Safe Water and AIDS Project (SWAP)	Pregnant women in Western Kenya	Maternity products: Free voucher to purchase products such as soap and water treatment supplies	Hirai et al., 2020 [77]	4
Kenya	Changamka’s mobile e-voucher	Pregnant women in Western Kenya	Maternal services, transportation and communication: Maternal voucher: either (1) fully paid e-voucher; (2) co-paid e-voucher for ANC, PNC, delivery, and transport; or (3) a control, namely health-related text messages and a helpline service	WHO 2013 * [78]	3
Cameroon	e-Voucher within Performance-based Financing (PBF)	Pregnant women in 2 Health Districts (Bali and Ndop) in the northwest region	Maternal services, transportation and communication: 5 e-vouchers that include (1) 4 ANC, (2) 1 delivery with five transportation vouchers, and (3) two-way communication and reminder messages through a mobile application	Nkangu et al., 2020 * [29]	5
Tanzania	‘Wired mothers’ cluster-randomized controlled trial	Pregnant women in Unguja and Zanzibar, Tanzania	Communication: A mobile phone voucher for direct two-way communication to contact the primary healthcare providers	Lund et al., 2012 * [79]	5
				Lund et al., 2014 * [80]	5

^1^ The quality of included studies was assessed by the criteria proposed by the National Health Service (NHS), UK, and adapted by Dixon-Woods et al. [30]. Each of five appraisal questions were scored 1 if yes and 0 if no. ^2^ MANEST: Innovations for Increasing Access to Integrated Safe Delivery; PMTCT (Prevention of mother-to-child transmission) and Newborn Care in Rural Uganda” (MANEST). ^3^ SMGL: Saving Mothers, Giving Life program by the United States government and other partners. * Studies that directly involve mobile phone system.

**Table 2:** Summary of included studies for Asia and the Middle East.

Country	Program	Target Population	Included Services under Voucher Scheme	Author, Year	Quality ^1^
Bangladesh	Maternal Health Voucher Scheme by Government of Bangladesh co-financed by World Health Organization (WHO), World Bank, GTZ, UK, European Community, Sweden, Germany, Canada, Netherlands, and UNFPA	Pregnant women with their first or second child in 33 subdistricts (Upazilas), either (1) universal for all pregnant women regardless of poverty status in 9 districts or (2) for targeted pregnant women based on eligibility criteria in 24 districts	Maternal services and transportation: Free voucher booklet for 3 ANC, facility or home-skilled delivery, 1 PNC, management of complications including C-section, transport cost of Taka 500 (USD 7; additional Taka 500 for referral), gift box of Taka 500 (USD 7) and cash payment of Taka 2000 (UDS 29)	Ahmed and Khan 2011a [81]	5
				Ahmed and Khan 2011b [82]	5
				Rob et al., 2011 [83]	5
				Hatt et al., 2010 [84]	5
				Koehlmoos et al., 2008 [85]	5
				Rob et al., 2010 [86]	5
				Schmidt et al., 2010 [87]	5
		Poor pregnant women with a household monthly income less than Taka 2500 (USD 29.19), and first pregnancy (second pregnancy eligible if using family planning methods) in 44 subdistricts (Upazilas)		Rahman et al., 2012 [88]	4
				Talukder et al., 2014 [89]	5
		Poor pregnant women with a household monthly income less than Taka 2500 (USD 29.19), and with a first or second child in 46 subdistricts (Upazilas)		Nguyen et al., 2012 [90]	5
				Keya et al., 2018 [91]	5
				Das and Nag 2018 [92]	4
		Poor pregnant women with a household monthly income less than Taka 2500 (USD 29.19), and with first or second child in 53 subdistricts (Upazilas)		Mahmood et al., 2019 [93]	5
				Mia et al., 2021 [94]	5
India	Sambhav Voucher Scheme funded by the U.S. Agency for International Development (USAID)	Pregnant women from the below poverty line (BPM) households in the selected districts (Agra District, Kanpur Nagar District, and Haridwar District)	Maternal services and transportation: A free voucher booklet for (1) 3 ANC visits (including maternal immunization), (2) delivery (normal, caesarean, and complicated), (3) 2 PNCs, (4) family planning, (5) reproductive tract infections or sexually transmitted infections (except Haridwar), (6) transportation subsidies	Donaldson et al., 2008 [95]	4
				IFPS Technical Assistant Project (ITAP), 2012 [96]	3
India	A cashless transport voucher scheme	Pregnant women from the BPM households, scheduled caste (SC) and scheduled tribe (ST) in the Purulia District of West Bengal	Transportation: A free transport voucher booklet for (1) transport from home to nearest health facility, (2) journey home, and (3) transport to higher-level center in case of referral	Mukhopadhyay et al., 2014 [97]	4
Pakistan	A 12-month maternal health voucher intervention in Dera Ghazi Khan City, funded by USAID	Pregnant women from the intervention neighborhood whose household income is below the national poverty line and who had no prior experience of delivery in a health facility	Maternal services and transportation: A voucher booklet was sold for USD 1.25, which included (1) 3 ANC visits (including complete blood count and ultrasound), (2) 1 PNC visit, (3) institutional delivery and (4) transportation	Agha S., 2011a [98]	5
Pakistan	The Jhang voucher scheme funded by Population Services International	Pregnant women in the two poorest quintiles of the Jiang District	Maternal services and transportation: A voucher booklet was sold for USD 1.20, which included (1) 3 ANC visits (including maternal tetanus immunization, complete blood count and ultrasound), (2) 1 PNC visit, (3) institutional delivery, (4) a postnatal family planning visit, and (5) transportation	Agha S., 2011b [99]	5
Pakistan	2 Transport voucher schemes under the Norwegian−Pakistani Partnership Initiative (NPPI) and family health insurance initiative (Sehat Sahulat Scheme)	Pregnant women living below the poverty line	Transportation: A voucher that covers the transportation costs	Mian et al., 2015 [100]	3
Laos	A supplementary voucher scheme under “Integrated Package of MNCH (maternal, newborns, child health) Services”	Pregnant women in the Heuamuang and Vienthong districts in Huaphan Province	Maternal services and transportation: A voucher for (1) free delivery services, and (2) subsidies for food and round-trip transportation for pregnant women who had at least 4 ANC visits	Heo et al., 2014 [101]	4
Myanmar	Ex-ante evaluation of the Maternal and Child Health Voucher Scheme (MCHVS)	Pregnant women with a low income	Maternal services and transportation: A voucher which includes (1) 4 ANC visits, (2) delivery, (3) PNC, and the cash for MCH service-related transportation, food and lodging	Myanmar Ministry of Health, et al., 2010 [102]	4
				Teerawattananon et al., 2014 [103]	1
				Kingkaew et al., 2016 [104]	5
	The pilot of MCHVS	Poor pregnant women in Yedarshey Township	Maternal services and transportation: A voucher booklet for (1) ANC, (2) delivery, (3) PNC, (4) infant immunization, and (5) subsidies for travel, food and accommodation	Pilasant et al., 2016 [105]	5
				Shwe et al., 2020 [106]	3
	Health technology assessment of MCHVS	Poor pregnant women living in hard-to-reach areas	Maternal services and transportation: A voucher booklet for (1) 4 ANC visits, (2) delivery, (3) 1 PNC visit, (4) 3–5 visits for immunization of the child, (5) subsidies for fees and transportation	Dabak et al., 2019 [107]	2
Indonesia	Vouchers for midwife services under Targeted Performance-Based Contracts for Midwives (TPC)	Poor pregnant women in ten districts in Java province, including Pemalang district (the study site)	Maternal services and transportation: A voucher booklet for (1) ANC, (2) PNC, (3) delivery, (4) referral to the hospital, (5) infant care, (6) birth control and (7) family health care	Tan, 2005 [108]	4
Cambodia	Voucher schemes initiated by the Belgian Technical Cooperation (BTC) and the Ministry of Health	Poor pregnant women in three health districts in Kampong Cham province	Maternal services and transportation: A voucher booklet for (1) 3 ANC visits, (2) delivery, (3) 1 PNC visit, and (4) transportation costs	Ir et al., 2008 [109]	4
				Ir et al., 2010 [110]	4
Cambodia	A reproductive health voucher scheme by the Cambodia Ministry of Health, with technical support from a consortium	Poor pregnant women in three pilot provinces (Kampong Thom, Kampot, and Prey Veng)	Maternal services and transportation: Vouchers for (1) ANC for up to 4 visits, (2) delivery, (3) PNC up to 6 weeks postpartum, (4) abortion services, (5) family planning counseling and services, and (6) transportation stipend based on kilometers travelled	Bellows et al., 2011 [111]	4
				Brody et al., 2013 [112]	5
Cambodia	Maternal health vouchers for services at public facilities	8 targeted schemes between 2007 to 2010 (including 4 that changed to a universal scheme) and 18 universal schemes started in 2008 (including 4 that changed from a targeted scheme)	Maternal services and transportation: Vouchers for (1) 4 ANC visits, (2) delivery, (3) 1 PNC visit, (4) reimbursement of transportation costs, and (5) fees for hospital referral covered by a health equity fund	Van de Poel et al., 2014 [113]	5
				Van de Poel et al., 2016 [114]	5
Yemen	Safe motherhood voucher scheme	Poor rural women in Lahj	Maternal services: A voucher for maternal health services (not specified) and 2 home visits by midwives (3 days after birth for checking the baby and 30 days after birth for family planning services)	Hyzam et al., 2020 [115]	5

^1^ The quality of included studies was assessed by the criteria proposed by the National Health Service (NHS), UK, and adapted by Dixon-Woods et al. [30]. Each of five appraisal questions were scored 1 if yes and 0 if no.

**Table 3:** Logic model for maternal health services or products via e-voucher programs.

Inputs	<Infrastructure and system>	1. Health information management system (routine monitoring of service statistics)	2. Electricity, water, telephone network, transportation, and ambulance	3. Health funds or insurance scheme (if possible)	4. **Mobile money platform** (for a co-pay voucher or e-voucher) or **mobile application**
	<Organization>	1. Voucher management agency (VMA)	2. Independent verification and evaluation agency	3. Contracted facilities	4. Interagency coordinating committee
		5. Partnership (e.g., NGOs and local government)	6. Project advisory group	7. Audit office for VMA	8. Steering committee
	<Staffing>	1. Study coordinator	2. (Community-based) voucher distributors and promoters/village health teams	3. Additional providers	4. Project manager
		5. Supervisor	6. Volunteers	7. Accredited social health activists	8. Quality improvement officer
		9. Community health workers/outreach workers and support (e.g., **a mobile phone** and non-monetary incentive)		10. Change champions/opinion leaders (e.g., mama ambassadors)	
	<Funding and resources>	1. Overall funding	2. Funds for pre-payment	3. Seed fund for facilities	4. Food, transportation, and accommodation subsidies
		5. Financial support for volunteers (travel and communication with midwives)	6. Technical support/assistance	7. Small premium to compensate facilities for administrative burden	8. Incentive for community health workers for identifying beneficiaries
		9. Conditional cash transfers combined with the voucher program (e.g., incentive for more than 4 ANC visits)			
	<Tools>	1. Voucher	2. Basic supplies and equipment (e.g., operating theaters)	3. Poverty grading tool/pre-defined questionnaire	4. Treatment guidelines, protocols, and training material
		5. Facility assessment tool (baseline and endline)	6. Printed birth plans	7. Mama kits for those delivered in facilities	8. Maternal death verbal autopsy tool (WHO)
		9. Theoretical frameworks	10. Criteria for selecting health centers/Accreditation checklist	11. Program information	
Activities	<Program design>	1. Formative research (e.g., consultative meetings, survey, key informant interview, focus group discussion, situation analysis, community survey, and literature review)	2. Estimation of birth rate/recruitment	3. Revenue planning and incentive sharing mechanism	4. Costing study/budgeting
		5. Benefits package design	6. Decision/negotiation on payment rates	7. Selection of pilot sites/target districts	8. Designing and printing vouchers, patient-held record, and branding logo
		9. Protocol development and adjustment	10. Development of eligibility criteria	11. Review on current standard MCH services	12. Field visits
		13. Health facility assessment	14. Selection of providers	15. Renovation of facilities	16. Design of health information management systems
		17. Needs assessment on behavior change communication		18. Development of a communication strategy (FGD with potential beneficiaries and interviews with physicians)	
	<Sensitization>	1. Sensitization of leaders	2. Community outreach (schools, church, traditional healers, and village chief)	3. Buy-in from political class	4. Mobilization of NGOs and community health volunteers
		5. Home visits for voucher promotion	6. Mobilization, behavior change communication and social marketing (e.g., radio, drama, posters, events, advertisements, and **multimedia campaigns**)		
	<Training, workshop and mentorship>	1. Health workers and midwives	2. Monthly group meetings with field coordinators	3. Community health workers, village health teams, accredited social health activists, etc. (**by mobile applications such as What’s App**)	
	<Payment>	1. Reimbursement	2. Verification of vouchers	3. Claims processing (**electronically or via SMS**)	4. Fraud control
		5. Adjustment of doctors’ salaries, reimbursement rates	6. Cost minimization evaluations	7. Transportation subsidy payment made by health centers	8. Accommodation of installment payment for those who cannot pay up front for vouchers (if necessary)
	<Implementation>	1. Preparation of facilities	2. Dissemination of project information	3. Engagement of traditional birth attendants	4. Pilot test
		5. Accreditation and contracting	6. Identification of beneficiaries, targeting, and screening (eligibility verification)	7. Registration/enrollment and data collection **via mobile phone**	8. Distribution (or sale) of vouchers
		9. Health education and promotion	10. Communication with providers	11. Comorbidity assessment	12. Stratification and/or randomization
		13. Project management and improvement within the context (including financial management)	14. Operation of one stop shops for HIV and family planning	15. Scale up planning and implementation	16. Cluster randomized controlled trials
		17. Quality assurance (e.g., hotline) and quality improvement (e.g., formation of quality improvement committees and incentives)			
	<Monitoring and evaluation>	1. Supervision (visits)	2. Audits, surveillance, and registry reviews (e.g., complications and deaths)	3. Pre-and post surveys, client satisfaction survey	4. Field testing of data collection forms
		5. Data collection and geo-mapping (via mobile phone)	6. Validation of claims by home visits and follow-up contacts	7. Follow-up with clients (**via mobile app**)	8. Verbal autopsy on maternal death
		9. Survey by an independent market research agency	10. Reporting	11. Ex-ante, on-going, and ex-post health technology assessment	12. Cost-effectiveness analysis
		13. Evaluation of midwives’ work	14. Monthly meeting and monitoring	15. Delisting of facilities if necessary	
Outputs	<Tools>	1. Manuals	2. Monthly reports	3. Reporting templates/data collection and entry forms	4. Anti-fraud policies
		5. Feasibility study report	6. Patient-held record	7. Guidelines for implementation	8. Costing questionnaires (pregnant women, new mothers, and providers)
		9. Number of supplies (e.g., mama kits) and equipment provided		10. Average number of equipment	
	<Staffing>	1. Number of staffs (e.g., village health teams) trained	2. Number of providers newly hired, trained, and mentored	3. Average number of human resources	4. Percentage of facilities with at least 1 doctor, nurse, or midwife
		5. Number of coordinators, community health workers, change champions, and midwives			
	<Payment>	1. Provider payment rates	2. Time from invoice to payment	3. Midwives’ monthly income	4. Unofficial payments made irrespective of voucher use
	<Implementation>	1. Number of meetings held	2. Number of marketing activities conducted (e.g., radio spot and drama skit)	3. Number of trainings	4. Timelines for planned activities
		5. Number of pregnant women received sensitization campaigns	6. Number of women able to activate GIS feature	7. Feedback into mobile platform	
	<Facilities>	1. Number of accredited facilities	2. Number of facilities improved (e.g., resource availability)	3. Number of districts covered under the program	4. Percentage of health areas with successful stratification and randomization
		5. Percentage of health centers excluded based on the criteria	6. Expansion of facilities	7. Withdrawal of accredited facilities due to overwhelming voucher clients	8. Number of facilities affiliated with community health workers
		9. Percentage of facilities with adequate infrastructure (electricity, running water, transportation, communication equipment, and maternal waiting shelter)	10. Percentage of facilities offering 24/7 services	11. Percentage of facilities with quality improvement activities (e.g., infrastructure, capital investment in equipment, supplies, staff, and patient amenities)	
	<Voucher use>	1. Number of vouchers distributed	2. Number of women who used vouchers	3. Number of vouchers sold/women who were sold vouchers	4. Number of vouchers re-deemed/used (for each service)
		5. Number of vouchers refunded	6. Number of fraud cases	7. Number of voucher claims submitted	8. Percentage of participants with successful enrollment within 6 months into trial
		9. Percentage of participants who complete follow-up at 8 months into trial	10. Percentage of participants who do not follow procedures as allocated	11. Percentage of contamination	12. Percentage of eligible women who received voucher booklets
		13. Average length of follow-up	14. Number of registered women	15. Number of poor pregnant women identified/contacted	16. Number of visits to the villages per year
		17. Increased demand for vouchers	18. Increased coverage for rural areas		
Outcomes (short-term)	<Service utilization—Delivery>	1. Proportion of estimated deliveries financed by the voucher	2. Percentage (number) of institutional deliveries (normal/c-section)	3. Rate of births at the enrolled facilities	4. Facility deliveries as a percentage of the expected number of births
		5. Number of deliveries by skilled birth attendants	6. Demand for attended delivery at the enrolled facilities	7. Number of deliveries by midwives	8. Percentage of home deliveries
	<Service utilization—ANC, PNC and others>	1. Proportion of women with (at least 4, 3, or 2) ANC visits (using vouchers)	2. Proportion of women with PNC visits within 7 days/48 h (using vouchers)	3. Increased equity in service utilization	4. Increased demand for ANC
		5. Number of services delivered by vouchers (ANC visits, ultrasound tests, PNC visits, and STI treatment)	6. Number of women who received key physical examinations (e.g., ultrasound, anemia exam, and urine test) complications management, blood transfusion, injection, or other drugs	7. Percentage of women with missed blood test and maternal immunization	8. Gestational age at first ANC visit
		9. Number of women who received postpartum examination and counseling before discharge	10. (Average) Number of ANC visits attended	11. Percentage of participants who adhered to the intervention	12. Number of women who completed the sequence of services
		13. Number of pregnant women who received first immunization using vouchers	14. Number of midwives’ services used	15. Percentage of women who sought care within 1 h from the onset of symptoms	16. Proportion of untreated complicated pregnancies
	<Staff and resources>	1. Effects on workload/overburdening	2. Overall satisfaction by providers	3. Challenges in identifying beneficiaries or distributing vouchers	4. Other challenges for providers
		5. Job satisfaction in terms of workload, salary, and staffing (frontline health workers, managers, and providers)	6. Providers’ attitudes towards voucher, accreditation, referral system and other healthcare needs	7. Motivation of providers for higher quality service	8. Changes in the resources used over time
	<Women’s experience>	1. Intention to recommend the voucher to a friend	2. Awareness of the voucher/number of women who heard of the program	3. Factors that facilitate/inhibit voucher use (pre-existing, distribution, and redemption factors)	4. Challenges for beneficiaries
		5. Reasons for using/not using vouchers (non-redemption)	6. Decision maker on the use of vouchers, health care service	7. Willingness-to-pay for satisfaction	8. Future decisions on—having children, average years to have next child, using the vouchers, place of delivery, and selection of healthcare provider
		9. Changes in fertility decision	10. Proportion of births prepared (chose where to deliver, saved money, and bought key materials)	11. Knowledge about possible dangers related to pregnancy, ANC or maternal immunization schedule	12. Knowledge, attitude and practice of MCH services (ANC, PNC, and delivery)
		13. Perceived barriers to MNCH services (opportunity cost of a round-trip to the nearest health facility, existing barriers—fees for drugs and consumables, transportation and distance, no time to visit due to work, dissatisfaction with facilities or equipment, staff or services, privacy, stigma, respect, waiting times, and quality)			
	<Quality>	1. Percentage of facilities that adequately considered medical history	2. Service waiting hours	3. Round-the-clock service availability	4. Percentage of facilities that adequately created rapport
		5. Availability of necessary service equipment, supplies, and logistics	6. Increased knowledge and skills of providers (e.g., life-threatening complications management)	7. Increased efficiency in service delivery	8. Satisfaction with the voucher/overall experience for delivery
		9. Possibility of clients being unattended due to large volume of voucher clients	10. Overall respect shown by the staff	11. Getting less attention than non-voucher clients	12. Providers indicating non-discriminatory attitudes
		13. Perceived quality by providers and women	14. Proportion of women receiving an acceptable quality of service (based on health facility assessment)	15. Perception on public health (contracted) facilities and quality of care	16. Maintaining privacy and confidentiality
	<Costs>	1. Out-of-pocket expenditure for maternal services, medicine, and transport	2. (Average) Total cost (per woman), reimbursement costs for each service, program management/administrative cost (e.g., data collection), cost per ANC and institutional delivery, and costs of goods and services	3. Direct medical cost, direct non-medical cost, and indirect cost (for ANC, delivery, PNC, complication, and vaccination)	4. Total cost for implementation and monitoring
		5. Incremental cost per institutional delivery, and maternal and newborn care	6. Percentage with additional emergency cost	7. Costs of setup and delivering the intervention	8. Protection from financial catastrophe due to pregnancy and delivery
		9. Net cost of program delivery per family	10. Weighted average costs for ANC, delivery, and PNC	11. Total cost of base wages	12. Total direct financial assistance to beneficiaries
	<Competition and governance>	1. Competition among providers	2. Increased client choices for providers	3. Institutionalization of accountability	4. Checks and balance mechanism
		5. Bypassing of low-quality service (re-warding services of higher quality)		6. Opportunities for learning and adapting to local settings	
Outcomes (long-term)	<Maternal and neonatal health outcomes>	1. Neonatal mortality rate	2. Infant mortality rate	3. Maternal mortality/number of maternal deaths (among those without seeking any health care)	4. Pre-discharge neonatal mortality
		5. Institutional perinatal mortality	6. (Institutional total and community) Still birth rate	7. (Institutional and community) Maternal mortality	8. Percentage of women with complications
		9. Percentage of postpartum hemorrhage, obstructed labor, and pre-eclampsia/eclampsia	10. Percentage of birth asphyxia, respiratory distress, prematurity	11. Deaths averted	12. DALYs averted
		13. (Mother’s and newborn) Life years saved per 1000 vouchers distributed		14. Reduction in malnutrition through empowerment of women	
	<Cost-effectiveness- Incremental Cost Effectiveness Ratio (ICER)>	1. Cost per death averted/(maternal, perinatal) life year saved	2. Cost per DALY averted		
	Sustainability of the program				
	Integration into health system				

Elements directly related to mobile platforms appear in bold text.

**Table 4:** Logic model for transportation via maternal health e-voucher programs.

Inputs	<Infrastructure>	**1. Telecommunication and telephone network**		2. Functioning road	
	<Staffing>	1. Transporters/drivers and medical team for ambulance		2. Community health workers and volunteers (e.g., village health teams)	
	<Tools and commodities>	1. Vehicles (e.g., ambulance)	2. Fuel	3. Guidelines for transport and referral	
	<Funding>	1. Transportation subsidies (**by mobile money**)		2. Financial contribution from the community	
Activities	<Program design>	1. Formative re-search (e.g., traveltime study), implementation research	2. Survey of transport providers	3. Sensitization of transporters	4. Organization of transport providers
		5. Ensuring licenses	6. Enlist the vehicles	7. Mobilization and sensitization of the community	8. Decisions on payment mechanism
		9. Resource mobilization from other partners	10. Creation of district transportation committees	11. Financial arrangement for nighttime or weekends, and long distance travel	
	<Implementation>	1. Presence of skilled birth attendance during transport	2. Introduction of the voucher to mothers	3. Identification of the target	4. Maintenance and disinfection of vehicles
		5. Provision of transport for other purposes (e.g., false labor pain or complications)		6. Payment/reimbursement (**by mobile money**)	
	<Monitoring and evaluation>	1. Regular meeting with stakeholders	2. Review of voucher charges	3. Supervision and mentorship	4. Authentication of the voucher by the attending doctor
	<Training and support>	1. Training of drivers, mothers, and staff	2. Training of frontline health workers for true labor pain and key danger signs	3. Administrative support	4. Male involvement and community dialogue
Outputs	<Tools>	1. Policy briefs	2. Regular reports		
	<Resource use>	1. Number of contracts signed with the transporters	2. Number of failed transactions on mobile money	3. Amount of payments made to transporters	4. Number of vehicles provided
Outcomes (short-term)	<Utilization>	1. Percentage of women using transportation vouchers	2. Number of mothers transported	3. Increased utilization of maternal health services	4. Bypassing resident health facilities
		5. Home deliveries and changing roles of TBAs		6. Percentage of institutional deliveries supported by the vouchers	
	<Women’s experience>	1. Number of women who said that the **mobile money transaction** process was easy	2. Number of women who are willing to save **mobile money for transport**	3. Increased fertility	4. Information gaps
	<Quality>	1. Quality of health services	2. Attitudes of health providers	3. Geographical inaccessibility	
	<Community involvement>	1. Acceptability by the community	2. Level of male involvement	3. Community suggestions for improvement	4. Community’s awareness level on maternal health
	<Potential issues>	1. Increased workload	2. Increased dependency	3. Implementation issues (e.g., voucher design and payment)	4. Need for scale-up/scalability (simplifying and complicating factors)
Outcomes (long-term)	Sustainability of the program				
	Integration into the existing health system				

Elements directly related to mobile platform appear in bold text.

**Table 5:** Logic model for communication via maternal health e-voucher programs (enabled by mobile phone credit voucher programs or maternal e-voucher programs involving mobile money).

Inputs	<Tools>	**1. Mobile application—audio and text message, and GIS feature for emergency**	2. Automated unidirectional **text messages**	3. **Mobile phone voucher** (credit)	4. Distribution of diagnostic equipment (e.g., electronic blood pressure metersand weighing scales)
Activities	<Program design>	1. Development of reminder message contents			
	<Implementation>	1. Two-way communication **via mobile phone**	2. Health facility selection and randomization	3. Training	4. Data entry for message generation
		5. Sending text messages and reminders **via mobile phone**		6. Quality control visits	
Outputs	<Transactions>	1. Percentage of participants who refused to respond	2. Percentage of participants whose mobile phone features failed to function	3. Number of women who received reminder text messages	
	<Systems monitoring>	1. Percentage of success in matching data sources	2. Percentage of functional communication network	3. Average time to collect data	
Outcomes (short-term)	<Maternal health service utilization>	1. Percentage of skilled delivery attendance by socioeconomic status	2. Percentage of women with ANC visits (1, 2, 3, 4, 5 or more)	3. Percentage of women with tetanus vaccination (1 or 2)	4. Percentage of women with preventive treatment for malaria
		5. Percentage of women for each gestational age at last ANC visit		6. Percentage of women with antepartum referral	
	<Acceptability>	1. Percentage of participants/providers who accept the use of **mobile health**			
Outcomes (long-term)	-				

Elements directly related to mobile platform appear in bold text.
